# Supplementary material for: Transcription shapes genome-wide histone acetylation patterns
Source: Nat Commun. 2021 Jan 11;12:210. doi: 10.1038/s41467-020-20543-z (PMC7801501; doi:10.1038/s41467-020-20543-z)
Supplement: Supplementary file 1 — Supplementary Information [file 41467_2020_20543_MOESM1_ESM.pdf]

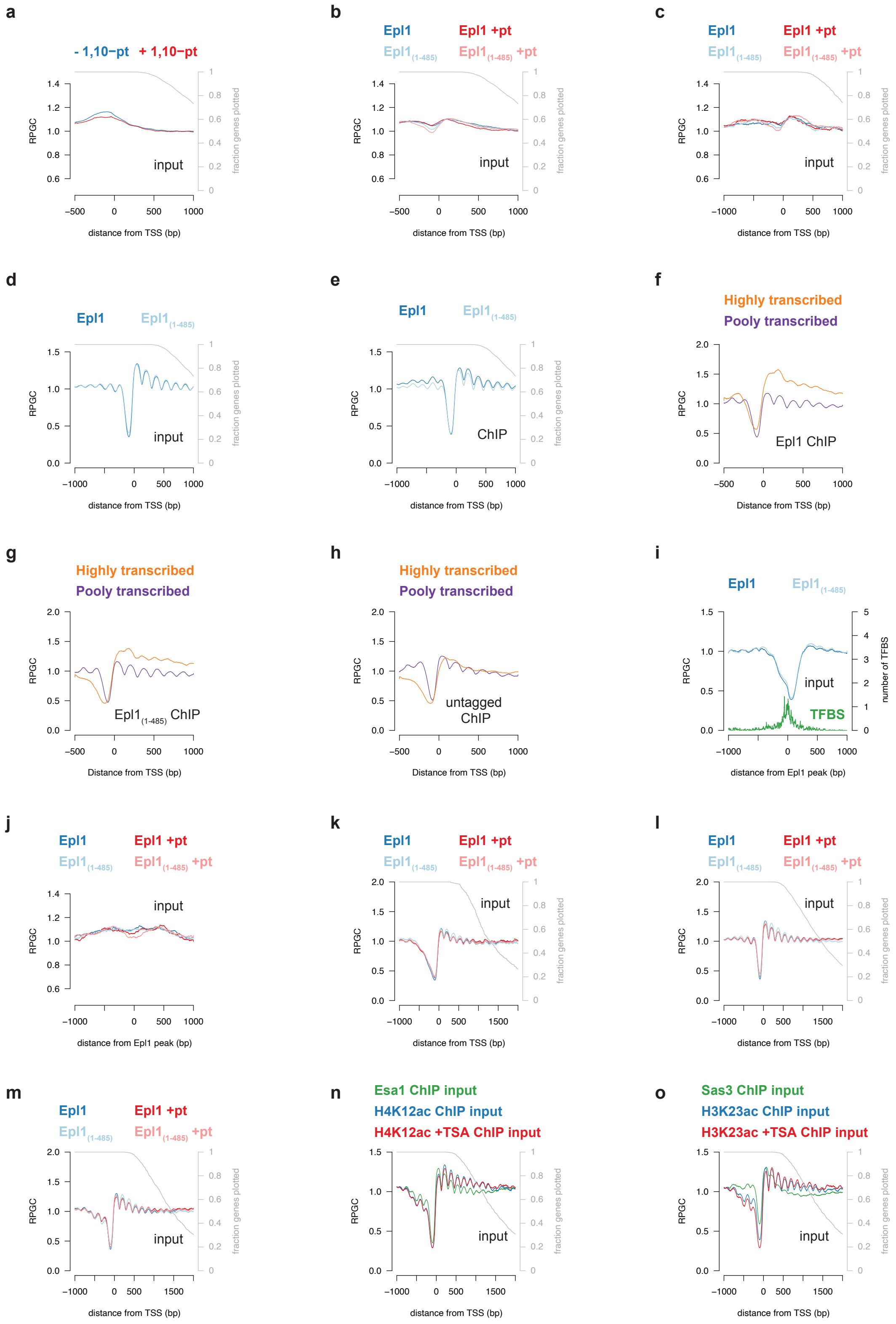

Supplementary Fig. 1 Average profiles of ChIP-seq inputs from data in Figures 1-3. Only data until the polyadenylation site (PAS) was included, and the grey line represents the fraction of genes still being plotted. RPGC, reads per genomic coverage; TSS, transcription start site; TFBS, transcription factor binding site[2].

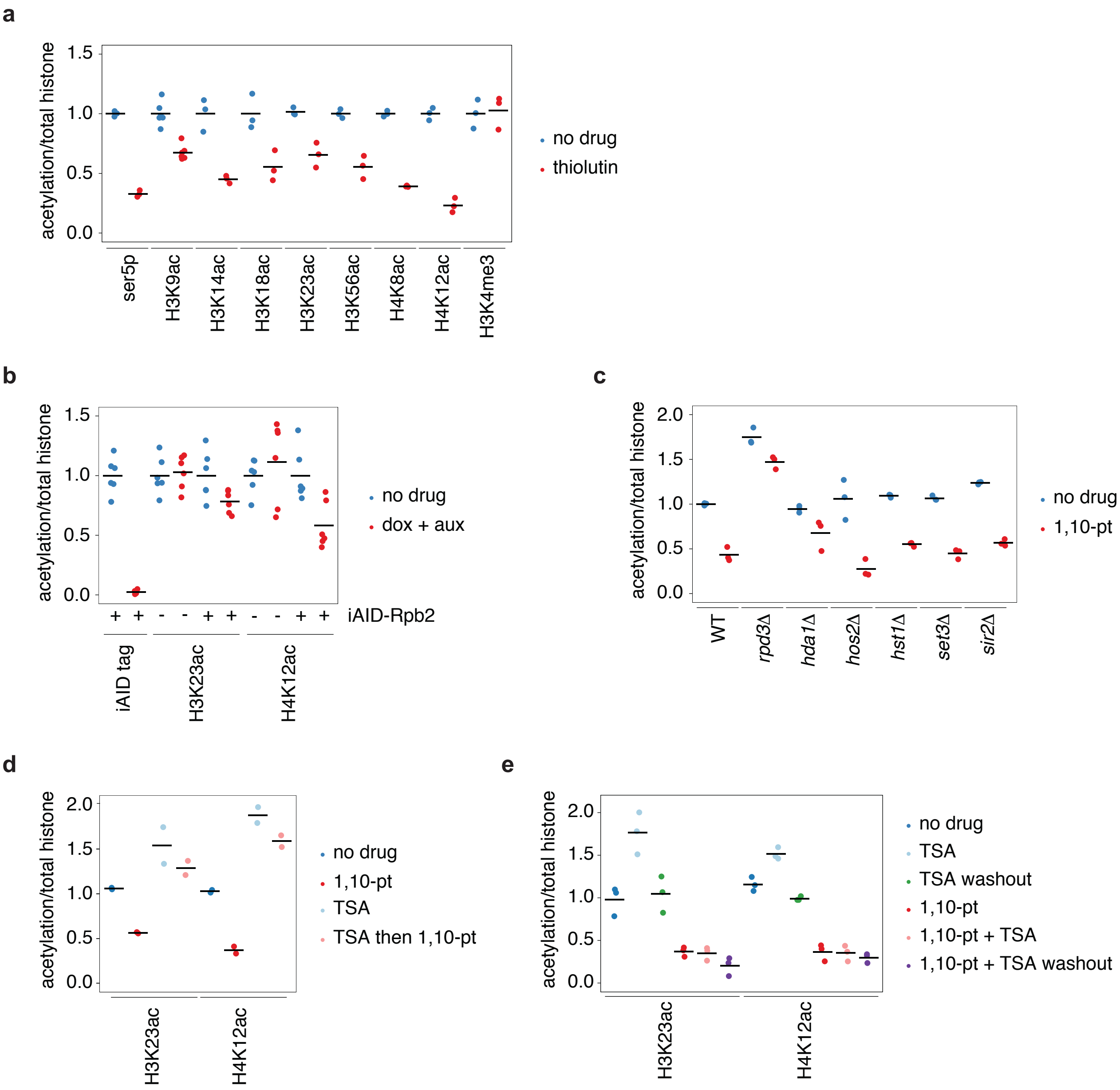

Supplementary Fig. 2 Characterization of histone deacetylation following transcription inhibition. **a.** Strip plot of histone PTM immunoblot signals normalized to histone H4 from three independent extracts from cells before and after treatment with thiolutin. Horizontal lines indicate the mean, with the vehicle control set to 1. **b.** Immunoblot signals, normalized to H3, of six independent whole cell extracts from the indicated strains after treatment with ethanol or auxin and doxycycline. **c.** Histone H4-normalized H4K12ac acetylation levels as determined by immunoblot of three independent extracts from wild type and deacetylase mutant strains treated with ethanol or 1,10-pt for 30 minutes. **d.** Cells were pretreated with the HDAC inhibitor trichostatin A (TSA) or DMSO for 15 minutes before a 30-minute treatment with 1,10-pt. Whole cell extracts were subjected to immunoblot analysis and strip plot representation of the two independent replicates is shown. Histone PTM signals were normalized to total histone H4 levels. **e.** Cells, pretreated with 1,10-pt or ethanol for 15 minutes, were subjected to a 15-minute treatment with TSA, before being washed into fresh media containing 1,10-pt or ethanol without TSA for 15 minutes. Whole cell extracts were subjected to immunoblot analysis and strip plot representation of the three independent replicates is shown. Histone PTM signals were normalized to total histone H4 levels.

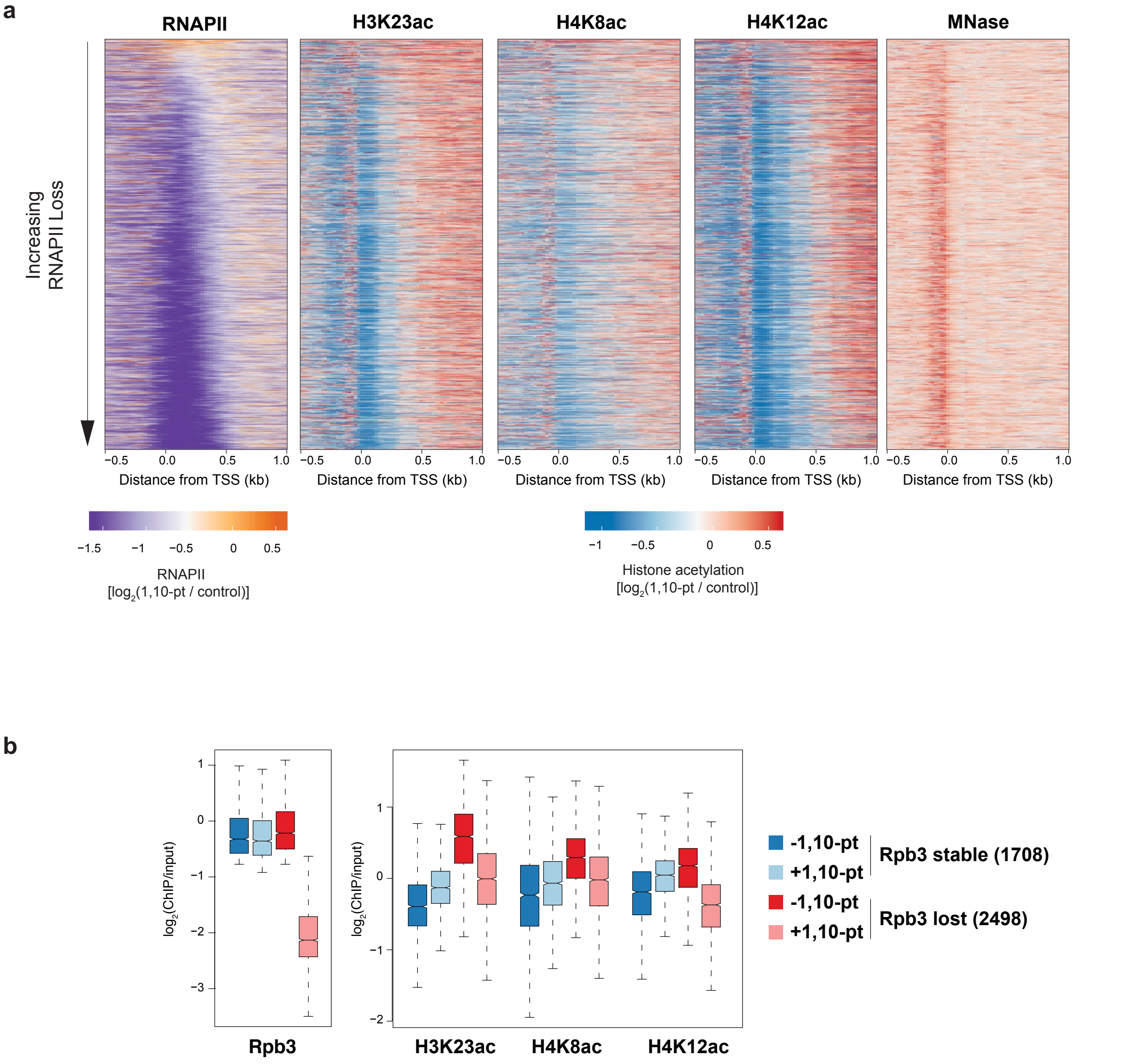

Supplementary Fig. 3 Histones are deacetylated following loss of RNAPII. **a.** Heatmaps representing the fold change (log_2) following transcription inhibition for RNAPII (Rpb3 ChIP-seq[3]), H3K23ac, H4K8ac, H4K12ac (MNase ChIP-seq), and nucleosome occupancy (MNase-seq), at 5133 genes aligned by the TSS. Heatmaps are ordered by the change in RNAPII occupancy with the colour scales below. **b.** Input normalized RNAPII (Rpb3 ChIP-seq) and nucleosome-normalized histone acetylation (MNase ChIP-seq) enrichments at transcribed nucleosomes with stable RNAPII and those that lost RNAPII upon treatment with 1,10-pt. Coloured boxes represent the interquartile range with a notch at the median, and whiskers extend 1.5 times the interquartile range.

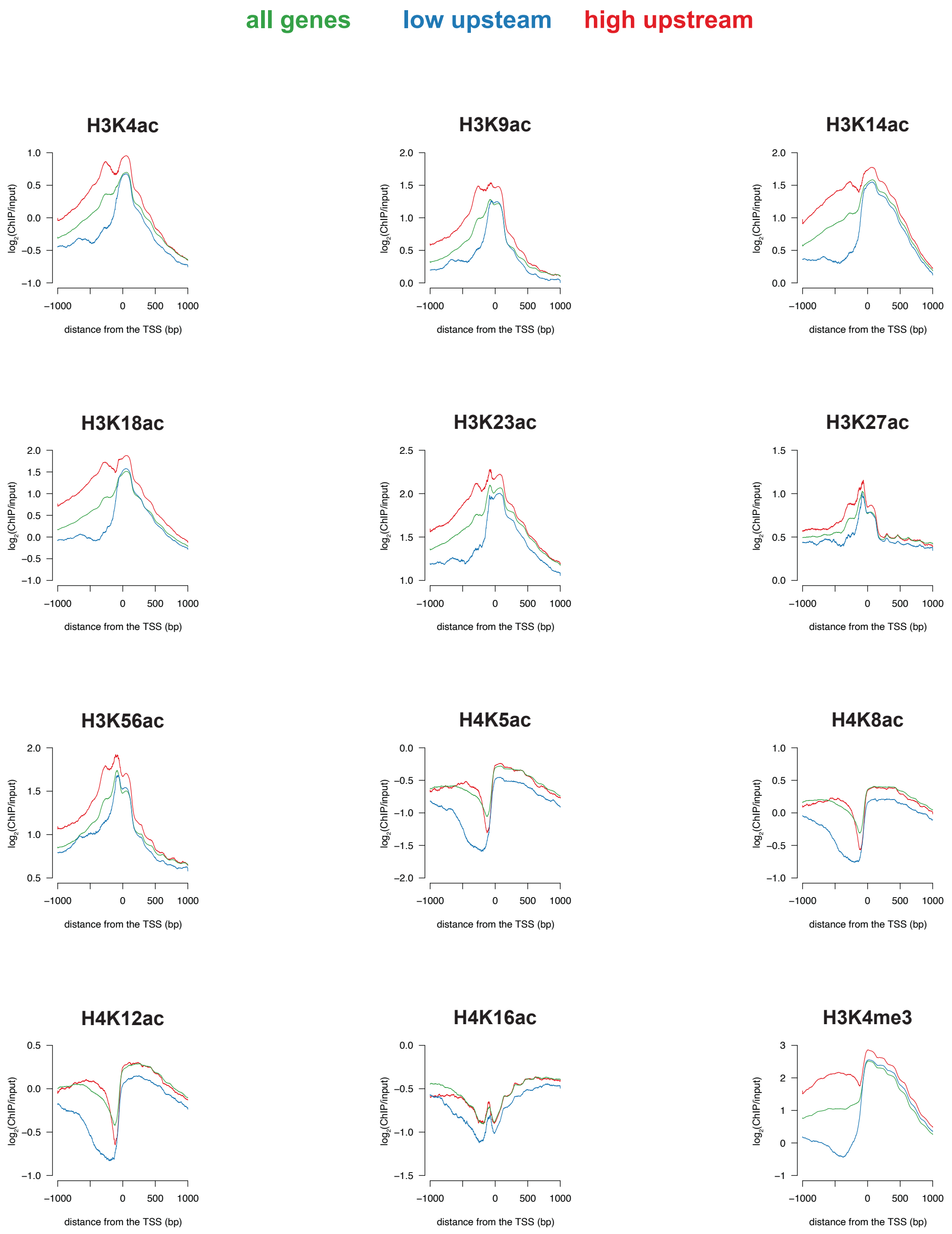

Supplementary Fig. 4 Acetylation upstream of promoters is associated with divergent transcription. The average signal relative to the TSS for the indicated histone PTMs[4] at all genes (green) or 832 transcribed genes that have low (blue) or high (red) upstream NET-seq signal. The ChIP data is presented as $\log_2$(ChIP/input) as nucleosome density is not consistent between the different gene bins.

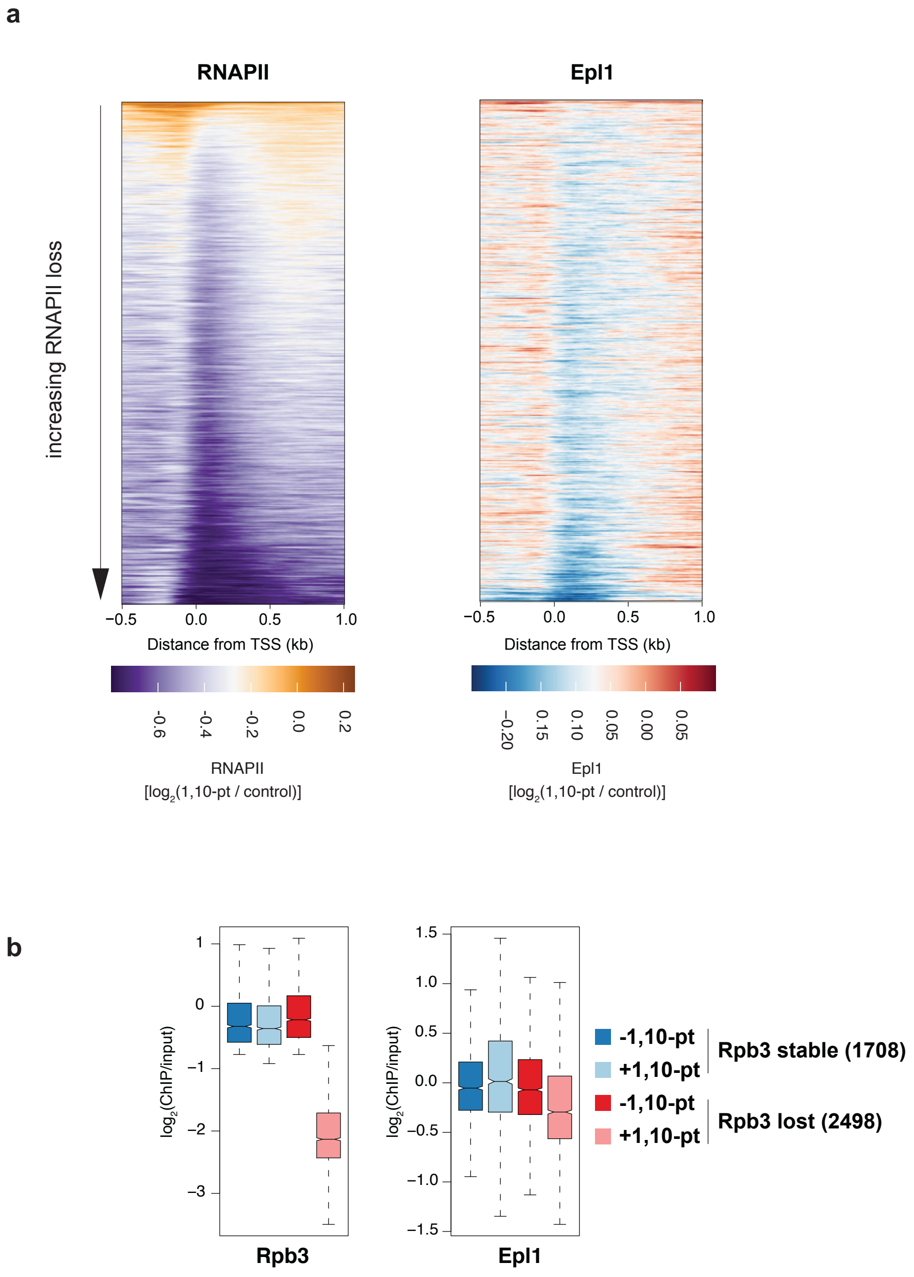

Supplementary Fig. 5 Epl1 and RNAPII are lost from similar regions following transcription inhibition. **a.** Heatmaps representing the fold change (log_2) following transcription inhibition for RNAPII (Rpb3 ChIP-seq[3]) and Epl1 ChIP-seq signal at 5207 genes aligned by the TSS. Heatmaps are ordered by the change in RNAPII occupancy with the colour scales below. **b.** Input normalized RNAPII (Rpb3 ChIP-seq[3]) and Epl1 ChIP-seq at transcribed nucleosomes with stable RNAPII and those that lost RNAPII upon treatment with 1,10-pt. Coloured boxes represent the interquartile range with a notch at the median, and whiskers extend 1.5 times the interquartile range.

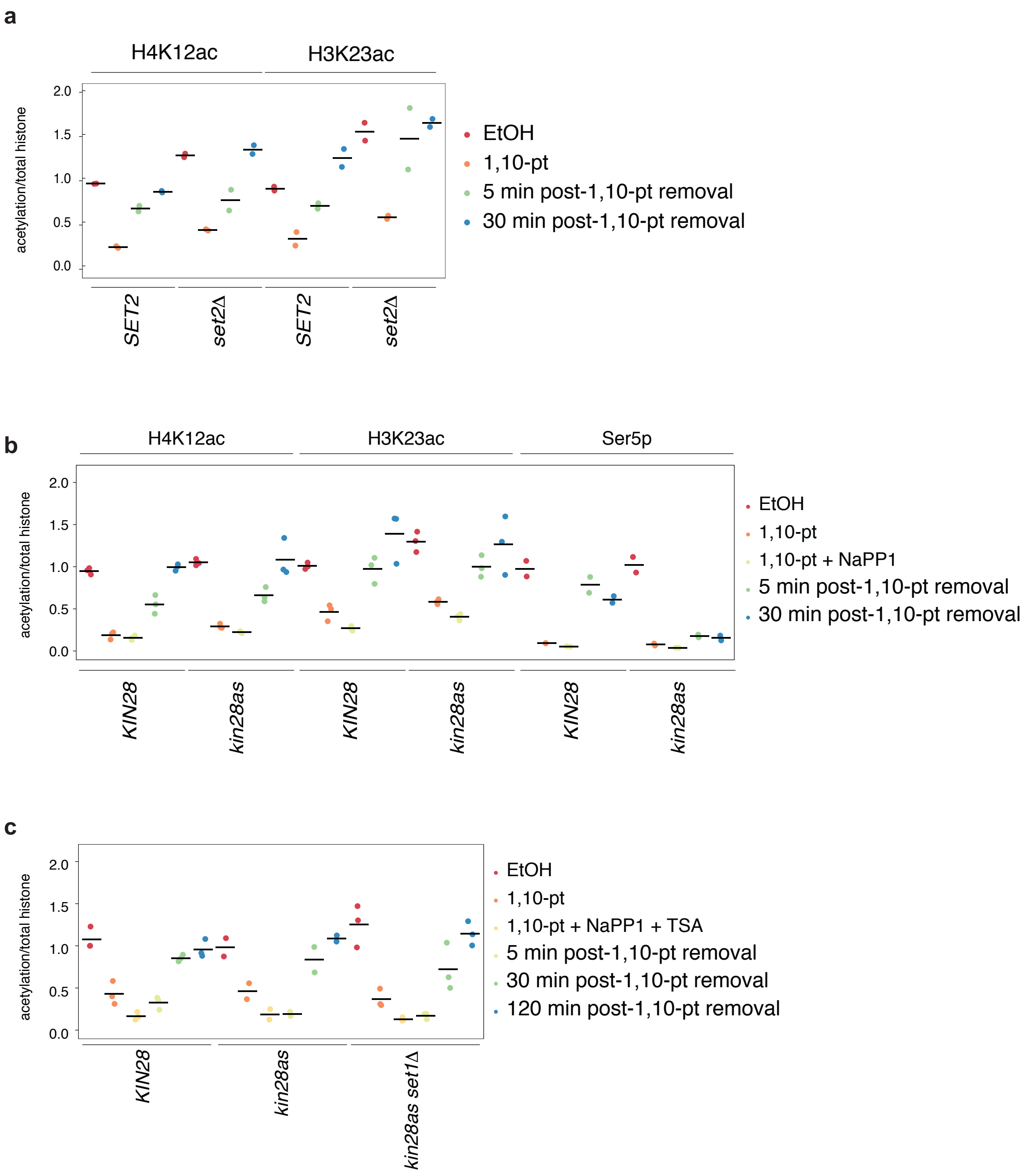

Supplementary Fig. 6 H3K36 methylation and RNAPII Ser5 phosphorylation are not required for reestablishment of acetylation following transcription inhibition. **a.** Wild type and *set2*Δ cells were treated with 1,10-pt for 30 minutes, followed by TSA treatment for an additional 30 minutes, before being washed into fresh media containing TSA. Samples were collected 5- and 30-minutes post-1,10-pt removal. Shown are strip plots of histone acetylation immunoblot signals normalized to histone H4 levels from two independent yeast whole cell extracts. **b.** and **c.** Strip plots of normalize immunoblot signals from three independent extracts from wild type and *kin28as* strains (**b**), or wild type, *kin28as*, *kin28as set1*Δ strains (**c**) treated with 1,10-pt for 30 minutes, followed by 1-NaPP1 for an additional 20 minutes, before being washed into fresh media containing only 1-NaPP1. Cells in (**c**) were also treated with TSA for an additional 30 minutes before being washed into fresh media containing TSA and 1-NaPP1, and were analyzed for H3K23ac levels.

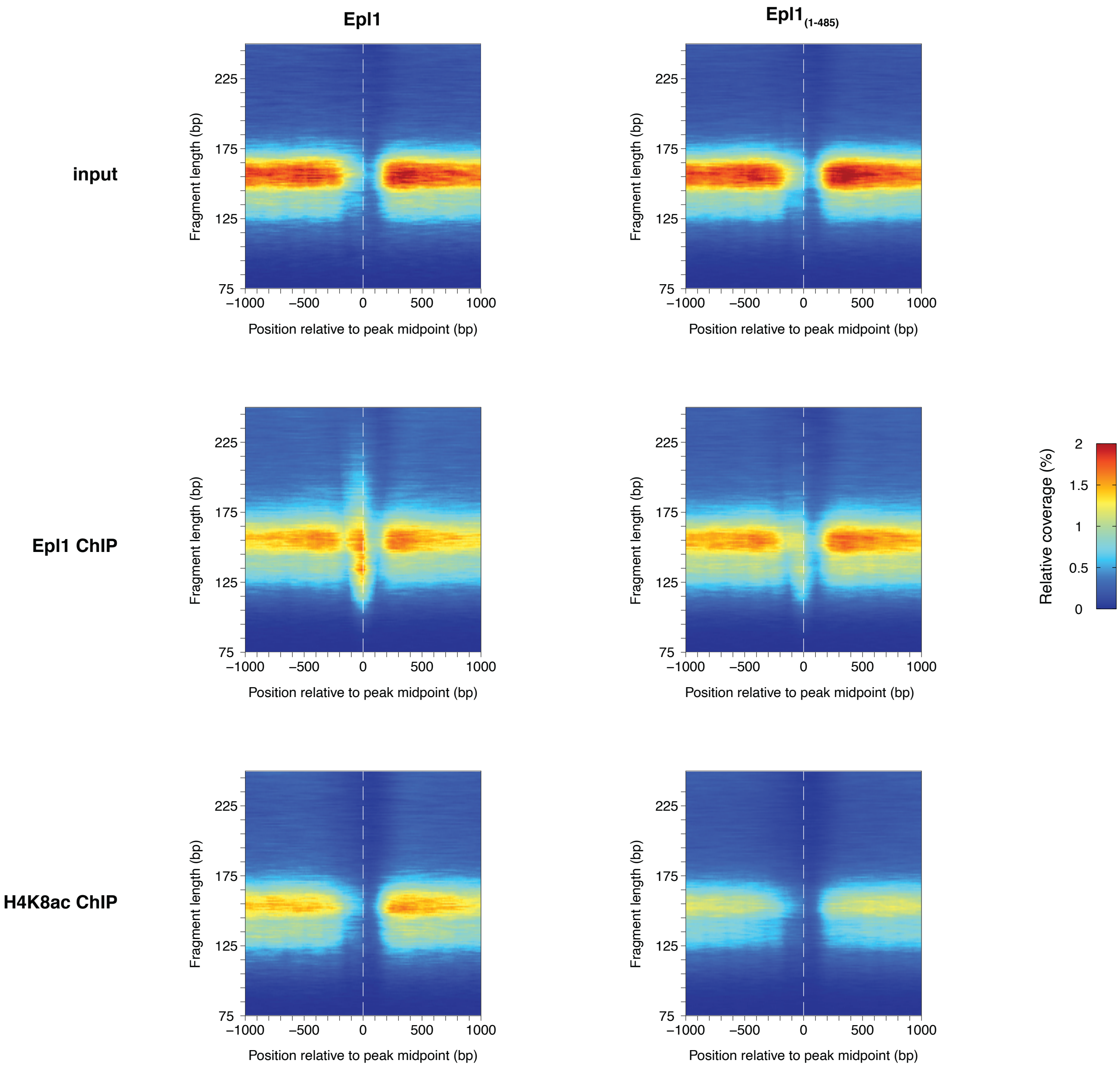

Supplementary Fig. 7 Promoter-localized Epl1 peaks are dependent on NuA4 and are not associated with acetylation. Two-dimensional occupancy plots of relative sequence fragment abundance, sequence fragment length, and sequence fragment position from input, Epl1, and H4K8ac ChIP-seq from MNase-digested chromatin, relative to the midpoint of 562 Epl1 peaks. Plot was generated using plot2DO[5] run with standard settings. The relative sequence read abundance is indicated as a heatmap with colour scale on the right, the sequence fragment length is plotted on the y-axis, and the position of sequence reads relative to the peak midpoint plotted on the x-axis.

a

Factor	Number of Peaks with Site	Fisher E-value	Adjusted p-value	Motif
Rap1	123	2.0×10^{-10}	2.0×10^{-14}	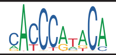
Aft2	99	1.7×10^{-5}	6.9×10^{-8}	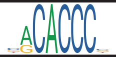
YPR022C	107	3.9×10^{-4}	1.5×10^{-3}	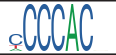
Tbs1	163	2.2×10^{-3}	1.2×10^{-5}	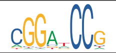
Adr1	122	5.0×10^{-3}	8.4×10^{-3}	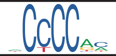
Met32	468	9.8×10^{-3}	1.4×10^{-2}	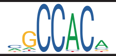
Phd1	28	1.3×10^{-2}	1.3×10^{-4}	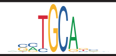
Ash1	32	5.2×10^{-2}	3.1×10^{-4}	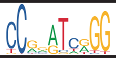
Skn7	279	8.6×10^{-2}	2.2×10^{-3}	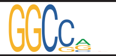

b

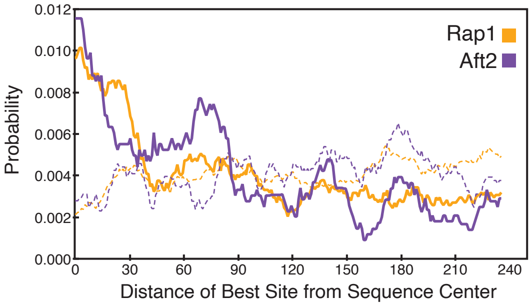

c

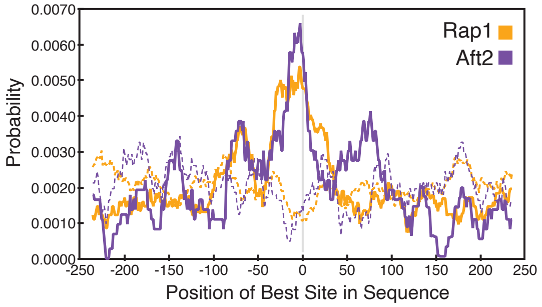

Supplementary Fig. 8 Analysis of DNA binding motifs at Epl1 NDR peak regions. Five hundred base pair regions around 562 Epl1 peaks were compared to 1958 NDRs depleted for Epl1 binding. Differential Enrichment for JASPAR non-redundant core fungi motifs[6] were calculated using the MEME suite[7]. JASPAR motifs with a Fisher E-value above 0.1 are shown in (**a**). For the top two hits, Rap1 and Aft2, CentriMo[8] was used to plot the distance from the best motif site to the Epl1 peak centre (**b**) and the motif probabilities around the best motif site for the regions containing target motifs (**c**). The solid regions represent the Epl1 peaks, while the dashed lines represent the negative control regions.

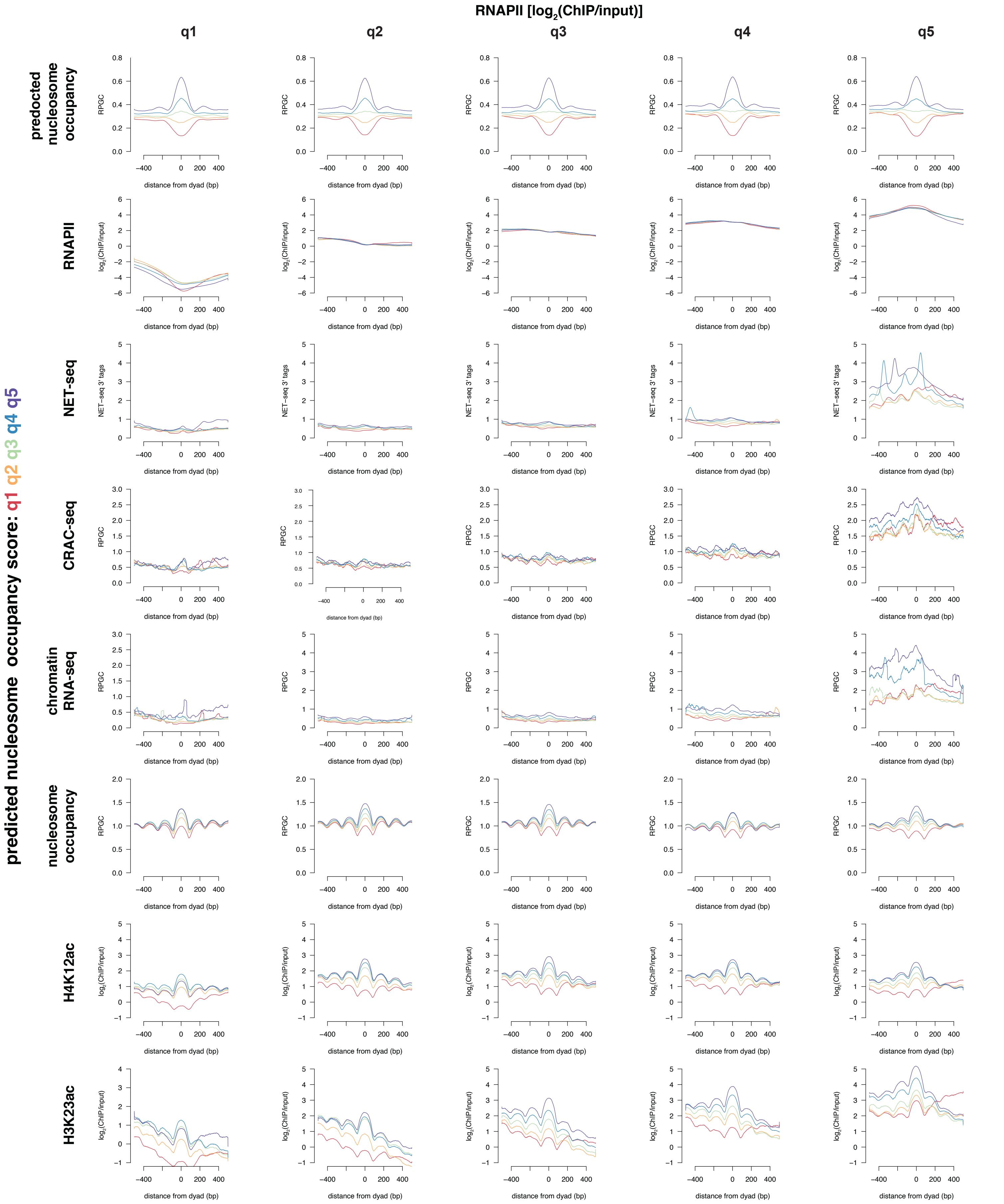

Supplementary Fig. 9 Histone acetylation is increased on nucleosomes predicted to impede RNAPII. Genic nucleosomes[4] divided into quintiles based on RNAPII occupancy [log_2(Rpb3/input) 50 bp upstream and downstream of nucleosome dyads], were further divided into quintiles based on predicted nucleosome occupancy[9] over the same region. Shown are profiles for predicted nucleosome occupancy, RNAPII (Rpb3 ChIP-seq[3]), NET-seq[10], CRAC-seq[11], chromatin-bound RNA-seq[10], nucleosome occupancy (MNase-seq from TSA-treated cells), and nucleosome-normalized H4K12ac and H3K23ac[12] ChIP-seq from TSA-treated cells upstream and downstream of the nucleosome dyad. RPGC, reads per genomic coverage.

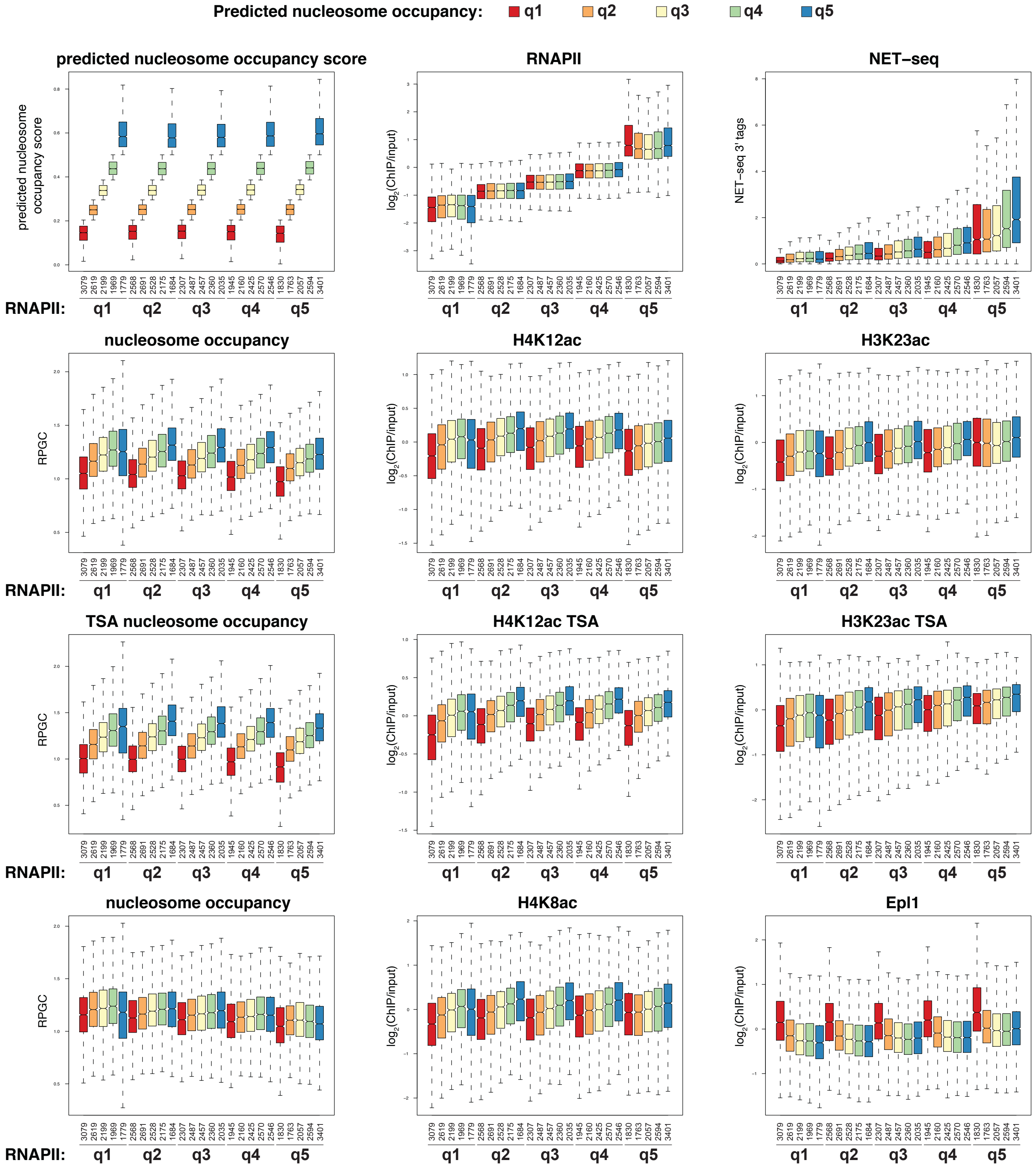

Supplementary Fig. 10 Histone acetylation is increased on nucleosomes predicted to impede RNAPII. Genic nucleosomes[4] divided into quintiles based on RNAPII occupancy [$\log_2$(Rpb3/input) 50 bp upstream and downstream of nucleosome dyads], were further divided into quintiles based on predicted nucleosome occupancy[9] over the same region. Shown are boxplots for predicted nucleosome occupancy, RNAPII (Rpb3 ChIP-seq[3]), NET-seq[10], nucleosome occupancy (MNase-seq), nucleosome-normalized H4K12ac, H3K23ac[12], and H4K8ac ChIP-seq, and Epl1 ChIP-seq for each quintile. The numbers below the x-axis are the number of nucleosomes in each bin. Coloured boxes represent the interquartile range with a notch at the median, and whiskers extend 1.5 times the interquartile range. RPGC, reads per genomic coverage.

Supplementary Table 1: Yeast Strains

YLH101	*his3Δ200 leu2Δ1 lys2-128δ ura3-52 trp1Δ63*
YLH220	*his3Δ200 leu2Δ1 lys2-128δ ura3-52 trp1Δ63 set1ΔHISMX6*
YLH353	*his3Δ200 leu2Δ1 lys2-128δ ura3-52 trp1Δ63 set2ΔTRP*
YLH404	*his3Δ200 leu2Δ1 lys2-128δ ura3-52 trp1Δ63 rpd3ΔTRP*
YLH556	*his3Δ200 leu2Δ1 lys2-128δ ura3-52 trp1Δ63 set3ΔKAN*
YLH559	*his3Δ200 leu2Δ1 lys2-128δ ura3-52 trp1Δ63 hos2ΔKAN*
YLH561	*his3Δ200 leu2Δ1 lys2-128δ ura3-52 trp1Δ63 sir2ΔKAN*
YLH562	*his3Δ200 leu2Δ1 lys2-128δ ura3-52 trp1Δ63 hst1ΔKAN*
YLH601	*his3Δ200 leu2Δ1 lys2-128δ ura3-52 trp1Δ63 KIN28*
YLH602	*his3Δ200 leu2Δ1 lys2-128δ ura3-52 trp1Δ63 kin28as*
YLH787	*his3Δ200 leu2Δ1 lys2-128δ ura3-52 trp1Δ63 bar1ΔKAN*
YLH827	*his3Δ200 leu2Δ1 lys2-128δ ura3-52 trp1Δ63 EPL1*$_{(1-485)}$*-6HA*
YLH828	*his3Δ200 leu2Δ1 lys2-128δ ura3-52 trp1Δ63 EPL1-6HA*
YLH945	*his3Δ200 leu2Δ1 lys2-128δ ura3-52 trp1Δ63 hda1ΔKAN*
YLH1020	*his3Δ200 leu2Δ1 lys2-128δ ura3-52 trp1Δ63 SSN6 pST1760 (YML119Wp-tTA (TetR-VP16) YML120Cp-TetR'-SSN6, TDH3p-OsTIR1, HIS3) RPB2-iAID (N-term) NAT*

Supplementary Table 2: Key Resources

H3K4me3	Abcam	ab1012, lot #1276040
H3K9ac	Custom antibody (Kimura et al., 2008)	CMA305
H3K14ac	Custom antibody (GeneScript)[1]	Affinity-purified rabbit polyclonal antibody
H3K18ac	Abcam	ab1191
H3K23ac	Active Motif	39131, lot # 1008001
H3K27ac	Active Motif	39133
H3K36ac	Abcam	ab177179, lot # GR205508
H3K56ac	Abcam	ab76307, lot # EPR996Y
H3K122ac	Abcam	ab33309, lot # GR3306851
H4K5ac	Millipore	07-327, lot # 2524676
H4K8ac	Abcam	ab45166, clone # EP1002Y
H4K12ac	Active Motif	39165, lot # 1008001
H4K16ac	Millipore	07-329, lot # 2506422
H3 (C-terminal region)	Custom antibody (GeneScript)[1]	Affinity-purified rabbit polyclonal antibody, Raised against scH3 peptide (CKDILARRLRGERS)
H4 (C-terminal region)	Abcam	ab31830, myeloma: Sp2/0-Ag14
HA	Roche	12CA5, lot # 11849700
3e8 (ser5p)	Millipore	04-1572, lot # 2585825

Supplementary Table 3: ChIP Normalization Factors

Sample	Normalization factor
Rpb3 ChIP +1,10-pt	0.399
H3K23ac ChIP +1,10-pt	0.940
H3K23ac ChIP +TSA.	1.24
H4K8ac ChIP +1,10-pt	0.934
H4K8ac ChIP in epl1$_{(1-485)}$ mutant	0.936
H4K8ac ChIP in epl1$_{(1-485)}$ mutant + 1,10-pt	0.814
H4K12ac ChIP +1,10-pt	0.938
H4K12ac ChIP +TSA	1.14
Epl1HA ChIP +1,10-pt	0.971
epl1$_{(1-485)}$HA ChIP	0.971
epl1$_{(1-485)}$HA CHIP +1,10-pt	0.926
epl1$_{(1-485)}$HA ChIP from MNase digested chromatin	0.972

Supplementary Table 4: ChIP-seq spike-ins (IDT gblocks)

ChIP-seq spike #1:<br>TAACTCTTTCGCTCCCTCATTCGTTCCTTCGCTAGTACCAGACCAACTGGTAATGGTAGC<br>GACCGGCGCTCAGCTGGAATTCCGCCGATACTGACGGGCTCCAGGAGTCGTCGCCACCAA<br>TCCCCATATGGAAACCGTCGATATTCAGCCATGTGCCTTCTTCCGCGTGCAGCAG
ChIP-seq spike in #2:<br>TAACTCTTTCGCTCCCTCATTCGTTCCTTCGCTAGTGCCGCCTTCATACTGCACCGGGCG<br>GGAAGGATCGACAGATTTGATCCAGCGATACAGCGCGTCGTGATTAGCGCCGTGGCCTGA<br>TTCATTCCCCAGCGACCAGATGATC

Supplementary References

1 Maltby, V. E. *et al.* Histone H3K4 demethylation is negatively regulated by histone H3 acetylation in Saccharomyces cerevisiae. *Proceedings of the National Academy of Sciences of the United States of America* **109**, 18505-18510, doi:Doi 10.1073/Pnas.1202070109 (2012).

2 MacIsaac, K. D. *et al.* An improved map of conserved regulatory sites for Saccharomyces cerevisiae. *BMC bioinformatics* **7**, 113, doi:10.1186/1471-2105-7-113 (2006).

3 Martin, B. J. E., Chruscicki, A. T. & Howe, L. J. Transcription Promotes the Interaction of the FAcilitates Chromatin Transactions (FACT) Complex with Nucleosomes in Saccharomyces cerevisiae. *Genetics* **210**, 869-881, doi:10.1534/genetics.118.301349 (2018).

4 Weiner, A. *et al.* High-resolution chromatin dynamics during a yeast stress response. *Molecular cell* **58**, 371-386, doi:10.1016/j.molcel.2015.02.002 (2015).

5 Beati, P. & Chereji, R. V. Creating 2D Occupancy Plots Using plot2DO. *Methods in molecular biology* **2117**, 93-108, doi:10.1007/978-1-0716-0301-7_5 (2020).

6 Khan, A. *et al.* JASPAR 2018: update of the open-access database of transcription factor binding profiles and its web framework. *Nucleic acids research* **46**, D1284, doi:10.1093/nar/gkx1188 (2018).

7 Ma, W., Noble, W. S. & Bailey, T. L. Motif-based analysis of large nucleotide data sets using MEME-ChIP. *Nature protocols* **9**, 1428-1450, doi:10.1038/nprot.2014.083 (2014).

8 Bailey, T. L. & Machanick, P. Inferring direct DNA binding from ChIP-seq. *Nucleic acids research* **40**, e128, doi:10.1093/nar/gks433 (2012).

9 Kaplan, N. *et al.* The DNA-encoded nucleosome organization of a eukaryotic genome. *Nature* **458**, 362-366, doi:10.1038/nature07667 (2009).

10 Harlen, K. M. *et al.* Comprehensive RNA Polymerase II Interactomes Reveal Distinct and Varied Roles for Each Phospho-CTD Residue. *Cell reports* **15**, 2147-2158, doi:10.1016/j.celrep.2016.05.010 (2016).

11 Milligan, L. *et al.* Strand-specific, high-resolution mapping of modified RNA polymerase II. *Molecular systems biology* **12**, 874, doi:10.15252/msb.20166869 (2016).

12 Martin, B. J. *et al.* Histone H3K4 and H3K36 Methylation Independently Recruit the NuA3 Histone Acetyltransferase in Saccharomyces cerevisiae. *Genetics* **205**, 1113-1123, doi:10.1534/genetics.116.199422 (2017).
