# Supplementary material for: Transcription shapes genome-wide histone acetylation patterns
Source: Nat Commun. 2021 Jan 11;12:210. doi: 10.1038/s41467-020-20543-z (PMC7801501; doi:10.1038/s41467-020-20543-z)
Supplement: Supplementary file 3 — Reporting Summary [file 41467_2020_20543_MOESM3_ESM.pdf]

nature research

Corresponding author(s): LeAnn Howe

Last updated by author(s): Oct 26, 2020

Reporting Summary

Nature Research wishes to improve the reproducibility of the work that we publish. This form provides structure for consistency and transparency in reporting. For further information on Nature Research policies, see Authors & Referees and the Editorial Policy Checklist .

Statistics

For all statistical analyses, confirm that the following items are present in the figure legend, table legend, main text, or Methods section.

n/a	Confirmed	
[ ]	[x]	The exact sample size (*n*) for each experimental group/condition, given as a discrete number and unit of measurement
[ ]	[x]	A statement on whether measurements were taken from distinct samples or whether the same sample was measured repeatedly
[ ]	[x]	The statistical test(s) used AND whether they are one- or two-sided<br>*Only common tests should be described solely by name; describe more complex techniques in the Methods section.*
[x]	[ ]	A description of all covariates tested
[x]	[ ]	A description of any assumptions or corrections, such as tests of normality and adjustment for multiple comparisons
[x]	[ ]	A full description of the statistical parameters including central tendency (e.g. means) or other basic estimates (e.g. regression coefficient) AND variation (e.g. standard deviation) or associated estimates of uncertainty (e.g. confidence intervals)
[x]	[ ]	For null hypothesis testing, the test statistic (e.g. *F*, *t*, *r*) with confidence intervals, effect sizes, degrees of freedom and *P* value noted<br>*Give P values as exact values whenever suitable.*
[x]	[ ]	For Bayesian analysis, information on the choice of priors and Markov chain Monte Carlo settings
[x]	[ ]	For hierarchical and complex designs, identification of the appropriate level for tests and full reporting of outcomes
[x]	[ ]	Estimates of effect sizes (e.g. Cohen's *d*, Pearson's *r*), indicating how they were calculated

Our web collection on statistics for biologists contains articles on many of the points above.

Software and code

Policy information about availability of computer code

Data collection	Data was collected using standard Illumina software for the NextSeq 500 and HiSeq 2500 platforms.

Data analysis

Analysis of ChIP-seq data
Adapter sequences were removed from paired end FASTQ files using cutadapt (version 1.83 – http://cutadapt.readthedocs.io/en/stable/), before aligning to the saccer3 genome using BWA (version 0.7.15-r1140)65. For analysis of epl1(1-485) data, reads of chrXII were removed from all data sets, as this chromosome appeared to be unstable in this mutant (1.5x coverage). Coverage tracks represent reads per genome coverage, calculated using the Java Genomics Toolkit version 1 (https://github.com/timpalpant/java-genomics-toolkit) scripts, ngs.BaseAlignCounts and wigmath.Scale. Log2 transformed ChIP over input tracks were calculated using the Java Genomics Toolkit and regions without signal in the input were removed to avoid division by 0. Replicates were pooled for subsequent analysis, and figures were generated in R.

Similar to other groups26,27, ChIP-seq datasets from 1,10-pt-treated or Epl1(1-485) cells were normalized to silent regions. The genome was divided into 250 bp bins, bins outside the interquartile range for coverage in the input were discarded, the 100 regions with the lowest Rpb3 signal were defined as silent regions, and these silent regions were used to normalized ChIP-seq datasets for cross-condition comparisons (Supplementary Table 3). We also added synthetic DNA spike-ins to our ChIP eluates and inputs (Supplementary Table 4), but this approach to normalization did not work well for all samples, possibly due to low coverage of the spike-ins in some samples.

Publicly available datasets
Publicly available datasets used in this paper are listed in Supplementary Table 5. FASTQ files from Weiner et al., 2015 were mapped to the saccer3 genome using BWA version 0.7.15-r114065. Reads were extended to 146 bp, and reads per genome coverage and log2 transformed ChIP over input files were calculated using deepTools version 3.0266.

Defining genome annotations
Yeast transcription start and end sites were downloaded from the supplemental files of Chereji et al., 201867. To identify active, non-

divergent, yeast promoters, genes in the lowest quintile of NET-seq signal over the first 500 bp downstream of the TSS were designated as non-transcribed. Unidirectional promoters were then defined as transcribed genes with the lowest quintile of NET-seq signal 100-600 bp upstream of the TSS (832 genes). RefSeq mm9 TSSs were downloaded from the UCSC Genome Browser (https://genome.ucsc.edu/). To identify mouse genes with active, non-divergent, promoters, transcribed genes were defined as those with greater than the median PRO-seq signal over 1 kb downstream of the TSS, and transcribed genes in the lowest quintile of PRO-seq signal upstream of the TSS were designated as having unidirectional promoters (3035 genes).

For transcribed nucleosomes classified by Rpb3 change upon 1,10-pt treatment (Supplementary Figures 3B and 5B), genome-wide nucleosome positions47 with Rpb3 signal greater than the median were classified as transcribed. Nucleosomes where Rpb3 changed by less than 10% were classified as “Rpb3 stable”, while those decreasing by at least 3x were classified as “Rpb3 lost”. Boxplots represent the 1st to 3rd quartiles, with whiskers extending to 1.5 times the interquartile range or to the extreme of the data. Notches are equal to +/- 1/58 IQR /sqrt(n), giving an approximation of the 95% confidence interval for the difference in 2 medians.

To find promoter peaks of Epl1, the Epl1 MNase ChIP-seq was compared to its input within the NDR for each gene67. Within each NDR, a smoothing spline was fit to the IP minus input signal (RPGC) and the peak position was selected. Peak positions with an IP minus input greater than 0.5 RPGC in the Epl1 ChIP-seq but not in the untagged control were selected as Epl1 peaks. NDRs in close proximity to tRNA genes or centromeres were removed from further analysis due to binding of Epl1 to these elements.

For motif analysis, Epl1 peaks were compared to 1958 NDR regions depleted for Epl1 binding (maximum IP minus input less than 0.1 RPGC). The 500 bp regions around peak centers were then input into the MEME-ChIP Differential Enrichment algorithm (https://www.nature.com/articles/nprot.2014.083?draft=collection) to find enriched motifs from the JASPAR non-redundant core fungi motifs (https://academic.oup.com/nar/article/46/D1/D260/4621338). For the top two hits, Rap1 and Aft2, CentriMo (https://academic.oup.com/nar/article/40/17/e128/2411117) was used to plot the distance from the best motif site to the Epl1 peak center and the motif probabilities around the best motif site for the regions containing target motifs.

Generating heatmaps and metaplots
Metaplot matrices centred on TSSs were generated using the sitepro script from the CEAS package 1.0.268 and matrices aligned to other features were produced using the visualization.MatrixAligner script from the Java Genomics Toolkit. Heatmap matrices were generated using deepTools69. Metaplots and heatmaps were generated using baseR and ComplexHeatmap version 1.2070 respectively. For 2D heatmaps, plot2DO (version 1) was used71

For manuscripts utilizing custom algorithms or software that are central to the research but not yet described in published literature, software must be made available to editors/reviewers. We strongly encourage code deposition in a community repository (e.g. GitHub). See the Nature Research guidelines for submitting code & software for further information.

Data

Policy information about availability of data

All manuscripts must include a data availability statement. This statement should provide the following information, where applicable:
- Accession codes, unique identifiers, or web links for publicly available datasets
- A list of figures that have associated raw data
- A description of any restrictions on data availability

Data generated for this manuscript were deposited in the NCBI Gene Expression Omnibus under the accession code GSE110287 [https://www.ncbi.nlm.nih.gov/geo/query/acc.cgi?acc=GSE110287]. Published datasets analyzed for this manuscript are detailed in Supplementary Table 5. Source data for Figures 1a and 1d, and Epl1 peak midpoints are provided in the Source Data file.

Field-specific reporting

Please select the one below that is the best fit for your research. If you are not sure, read the appropriate sections before making your selection.

☒ Life sciences ☐ Behavioural & social sciences ☐ Ecological, evolutionary & environmental sciences

For a reference copy of the document with all sections, see nature.com/documents/nr-reporting-summary-flat.pdf

Life sciences study design

All studies must disclose on these points even when the disclosure is negative.

Sample size	Sample sizes for immunoblots were determined by the maximum number of samples that could be run on a single blot. Sample sizes for ChIP-seq experiments was limited to two replicates due to financial constraints.
Data exclusions	For analysis of epl1(1-485) data reads of chrXII were removed from all data sets as this chromosome appeared to be unstable in this mutant (1.5x coverage).
Replication	The reproducibility for all sequencing data and analyses was confirmed by two independent experiments.
Randomization	All experiments were performed with large numbers of isogenic cells and thus randomization was not required.
Blinding	Blinding was not required because the data analysis pipelines used prevent human bias.

Reporting for specific materials, systems and methods

We require information from authors about some types of materials, experimental systems and methods used in many studies. Here, indicate whether each material, system or method listed is relevant to your study. If you are not sure if a list item applies to your research, read the appropriate section before selecting a response.

Materials & experimental systems

n/a	Involved in the study
☐	☒ Antibodies
☐	☒ Eukaryotic cell lines
☒	☐ Palaeontology
☒	☐ Animals and other organisms
☒	☐ Human research participants
☒	☐ Clinical data

Methods

n/a	Involved in the study
☐	☒ ChIP-seq
☒	☐ Flow cytometry
☒	☐ MRI-based neuroimaging

Antibodies

Antibodies used

Note: histones H3 and H4 are extremely well conserved between yeast and humans. Indeed all PTM sites analyzed in this manuscript are identical between yeast and human histones. The C-terminus of H3 is divergent between yeast and human and thus we generated a custom yeast-specific antibody for this site.
anti-H3K4me3 rabbit antibody (Abcam ab1012, lot #1276040)
anti-H3K9ac rabbit antibody custom antibody (Kimura et al., 2008, CMA305)
anti-H3K14ac custom rabbit antibody (GeneScript, affinity-purified)
anti-H3K18ac rabbit antibody (Abcam ab1191)
anti-H3K23ac rabbit antibody (Active Motif 39131, lot # 1008001)
anti-H3K27ac rabbit antibody (Active Motif 39133)
anti-H3K36ac rabbit antibody (Abcam ab177179, lot # GR205508)
anti-H3K56ac rabbit antibody (Abcam ab76307, lot # EPR996Y)
anti-H3K122ac rabbit antibody (Abcam ab33309, lot # GR3306851)
anti-H4K5ac rabbit antibody (Millipore 07-327, lot # 2524676)
anti-H4K8ac rabbit antibody (Abcam ab45166, clone # EP1002Y)
anti-H4K12ac rabbit antibody (Active Motif 39165, lot # 1008001)
anti-H4K16ac rabbit antibody (Millipore 07-329, lot # 2506422)
anti-H3 custom rabbit antibody (GeneScript, raised against CKDILARRLRGERS)
anti-H4 mouse antibody (Abcam ab31830, myeloma: Sp2/0-Ag14)
anti-HA rat antibody (Roche 12CA5, lot # 11849700)
anti-Rpb1 ser5p mouse antibody (3e8, Millipore 04-1572, lot # 2585825)

Validation

anti-H3K4me3: immunoblot signal specifically disrupted by H3K4me3 peptides and not H3K4me2, me1, or me0 peptides.
anti-H3K9ac: immunoblot signal reduced greater than 80% in gcn5 deletioin
anti-H3K14ac: immunoblot signal reduced greater than 90% in ada2sas3 deletion
anti-H3K18ac: immunoblot signal reduced greater than 90% in gcn5 deletion
anti-H3K23ac: immunoblot signal reduced greater than 95% in H3K23R mutant
anti-H3K56ac: immunoblot signal reduced greater than 90% in rtt109 mutant
anti-H3K122ac: immunoblot signal at expected size observed in WCEs.
anti-H4K5ac: immunoblot signal reduced greater than 90% in esa1-ts strain grown at 37 degrees C
anti-H4K8ac: immunoblot signal reduced greater than 90% in esa1-ts strain grown at 37 degrees C
anti-H4K12ac: immunoblot signal reduced greater than 90% in esa1-ts strain grown at 37 degrees C
anti-H4K16ac: immunoblot signal at expected size observed in WCEs.
anti-H3: immunoblot signal at expected size observed in WCEs and using recombinant expressed histone H3.
anti-H4: immunoblot signal at expected size observed in WCEs and using recombinant expressed histone H4.
anti-HA: immunoblot, ChIP-qPCR, and ChIP-seq confirmed specific reactivity to strains with HA-tagged proteins and little to no reactivity to untagged strains.
anti-Rpb1 ser5p: immunoblot signal at expected size

Eukaryotic cell lines

Policy information about cell lines

Cell line source(s)

All yeast strains used in this study are listed in Supplementary Table 1. The mESC line used is described in Chen et al., 2018 (PMID: 29229671) and was derived from TT2 cells. TT2 cells were established from an F1 embryo between a C57BL/6 female and a CBA male as F1/1 cells by Suda et al., 1987 (PMID: 2832150).

Authentication	All yeast strains were verified by PCR of genomic DNA. For the strains expressing tagged proteins, the strains were verified first by PCR of genomic DNA and immunoblot analysis.
Mycoplasma contamination	All cell lines tested are free for mycoplasma contamination.
Commonly misidentified lines (See ICLAC register)	No commonly misidentified cell lines were used in the study.

ChIP-seq

Data deposition

☒ Confirm that both raw and final processed data have been deposited in a public database such as GEO.

☒ Confirm that you have deposited or provided access to graph files (e.g. BED files) for the called peaks.

Data access links
May remain private before publication.

https://www.ncbi.nlm.nih.gov/geo/query/acc.cgi?acc=GSE110287

Files in database submission

GSM2985387_pt15_rep1_input_coverage_per_mil_frag.wig
GSM2985388_tp0_rep1_H4K12ac_coverage_per_mil_frag.wig
GSM2985389_pt15_rep1_H4K12ac_coverage_per_mil_frag.wig
GSM2985390_tsa15_rep1_H4K12ac_coverage_per_mil_frag.wig
GSM2985391_pt15_rep1_H3K23ac_coverage_per_mil_frag.wig
GSM2985392_pt15_rep2_Input_coverage_per_mil_frag.wig
GSM2985393_tp0_rep2_H4K12ac_coverage_per_mil_frag.wig
GSM2985394_pt15_rep2_H4K12ac_coverage_per_mil_frag.wig
GSM2985395_tsa15_rep2_H4K12ac_coverage_per_mil_frag.wig
GSM2985396_pt15_rep2_H3K23ac_coverage_per_mil_frag.wig
GSM2985412_EPL1HA6_rep1_tp0_100uMNase_H4K8ac_coverage_per_mil_frag.wig
GSM2985413_EPL1HA6_rep2_tp0_100uMNase_H4K8ac_coverage_per_mil_frag.wig
GSM2985414_EPL1HA6_rep1_tp_pt15_100uMNase_H4K8ac_coverage_per_mil_frag.wig
GSM2985415_EPL1HA6_rep2_tp_pt15_100uMNase_H4K8ac_coverage_per_mil_frag.wig
GSM2985416_EPL1HA6_rep1_tp0_100uMNase_Input_coverage_per_mil_frag.wig
GSM2985417_EPL1HA6_rep2_tp0_100uMNase_Input_coverage_per_mil_frag.wig
GSM2985418_EPL1HA6_rep1_tp_pt15_100uMNase_Input_coverage_per_mil_frag.wig
GSM2985419_EPL1HA6_rep2_tp_pt15_100uMNase_Input_coverage_per_mil_frag.wig
GSM2985433_EPL1HA6_rep1_tp0_Sonicated_HA_IP_coverage_per_mil_frag.wig
GSM2985434_EPL1HA6_rep2_tp0_Sonicated_HA_IP_coverage_per_mil_frag.wig
GSM2985435_EPL1HA6_rep1_tp_pt15_Sonicated_HA_IP_coverage_per_mil_frag.wig
GSM2985436_EPL1HA6_rep2_tp_pt15_Sonicated_HA_IP_coverage_per_mil_frag.wig
GSM2985437_EPL1HA6_rep1_tp0_Sonicated_Input_coverage_per_mil_frag.wig
GSM2985438_EPL1HA6_rep2_tp0_Sonicated_Input_coverage_per_mil_frag.wig
GSM2985439_EPL1HA6_rep1_tp_pt15_Sonicated_Input_coverage_per_mil_frag.wig
GSM2985440_EPL1HA6_rep2_tp_pt15_Sonicated_Input_coverage_per_mil_frag.wig
GSM4100666_EPL1_rep1_tp0_100uMNase_HA_IP_coverage_per_mil_frag.wig
GSM4100667_epl1.485_rep1_tp0_100uMNase_HA_IP_coverage_per_mil_frag.wig
GSM4100668_epl1.485_rep1_tp0_100uMNase_Input_coverage_per_mil_frag.wig
GSM4100669_epl1.485_6HA_rep1_tp0_Sonicated_HA_IP_coverage_per_mil_frag.wig
GSM4100670_epl1.485_6HA_rep1_tp0_Sonicated_Input_coverage_per_mil_frag.wig
GSM4100671_epl1.485_6HA_rep1_tp_pt15_Sonicated_HA_IP_coverage_per_mil_frag.wig
GSM4100672_epl1.485_6HA_rep1_tp_pt15_Sonicated_Input_coverage_per_mil_frag.wig
GSM4100673_epl1.485_6HA_rep2_tp0_Sonicated_HA_IP_coverage_per_mil_frag.wig
GSM4100674_epl1.485_6HA_rep2_tp0_Sonicated_Input_coverage_per_mil_frag.wig
GSM4100675_epl1.485_6HA_rep2_tp_pt15_Sonicated_HA_IP_coverage_per_mil_frag.wig
GSM4100676_epl1.485_6HA_rep2_tp_pt15_Sonicated_Input_coverage_per_mil_frag.wig
GSM4849495_epl1.485HA6_rep1_tp0_100uMNase_H4K8ac_coverage_per_mil_frag.wig
GSM4849496_epl1.485HA6_rep2_tp0_100uMNase_H4K8ac_coverage_per_mil_frag.wig
GSM4849497_epl1.485HA6_rep1_pt15_100uMNase_H4K8ac_coverage_per_mil_frag.wig
GSM4849498_epl1.485HA6_rep2_pt15_100uMNase_H4K8ac_coverage_per_mil_frag.wig
GSM4849499_epl1.485HA6_rep2_tp0_100uMNase_Input_coverage_per_mil_frag.wig
GSM4849500_epl1.485HA6_rep1_pt15_100uMNase_Input_coverage_per_mil_frag.wig
GSM4849501_epl1.485HA6_rep2_pt15_100uMNase_Input_coverage_per_mil_frag.wig
GSM4849502_Untagged_100uMNase_HA_IP_coverage_per_mil_frag.wig
GSM4849503_Untagged_100uMNase_Input_coverage_per_mil_frag.wig
GSM4850569_tp0_rep1_H3K23ac_coverage_per_mil_frag.wig
GSM4850570_tp0_rep1_Input_coverage_per_mil_frag.wig
GSM4850571_tp0_rep2_H3K23ac_coverage_per_mil_frag.wig
GSM4850572_tp0_rep2_Input_coverage_per_mil_frag.wig

GSM4850573_tsa15_rep1_H3K23ac_coverage_per_mil_frag.wig
GSM4850574_tsa15_rep1_Input_coverage_per_mil_frag.wig
GSM4850575_tsa15_rep2_H3K23ac_coverage_per_mil_frag.wig
GSM4850576_tsa15_rep2_Input_coverage_per_mil_frag.wig
EPL1_rep1_tp0_100uMNase_HA_IP_R1.fastq
EPL1_rep1_tp0_100uMNase_HA_IP_R2.fastq
epl1.485_6HA_rep1_tp_pt15_Sonicated_HA_IP_R1.fastq
epl1.485_6HA_rep1_tp_pt15_Sonicated_HA_IP_R2.fastq
epl1.485_6HA_rep1_tp_pt15_Sonicated_Input_R1.fastq
epl1.485_6HA_rep1_tp_pt15_Sonicated_Input_R2.fastq
epl1.485_6HA_rep1_tp0_Sonicated_HA_IP_R1.fastq
epl1.485_6HA_rep1_tp0_Sonicated_HA_IP_R2.fastq
epl1.485_6HA_rep1_tp0_Sonicated_Input_R1.fastq
epl1.485_6HA_rep1_tp0_Sonicated_Input_R2.fastq
epl1.485_6HA_rep2_tp_pt15_Sonicated_HA_IP_R1.fastq
epl1.485_6HA_rep2_tp_pt15_Sonicated_HA_IP_R2.fastq
epl1.485_6HA_rep2_tp_pt15_Sonicated_Input_R1.fastq
epl1.485_6HA_rep2_tp_pt15_Sonicated_Input_R2.fastq
epl1.485_6HA_rep2_tp0_Sonicated_HA_IP_R1.fastq
epl1.485_6HA_rep2_tp0_Sonicated_HA_IP_R2.fastq
epl1.485_6HA_rep2_tp0_Sonicated_Input_R1.fastq
epl1.485_6HA_rep2_tp0_Sonicated_Input_R2.fastq
epl1.485_rep1_tp0_100uMNase_HA_IP_R1.fastq
epl1.485_rep1_tp0_100uMNase_HA_IP_R2.fastq
epl1.485_rep1_tp0_100uMNase_Input_R1.fastq
epl1.485_rep1_tp0_100uMNase_Input_R2.fastq
epl1.485HA6_rep1_pt15_100uMNase_H4K8ac_R1.fastq
epl1.485HA6_rep1_pt15_100uMNase_H4K8ac_R2.fastq
epl1.485HA6_rep1_pt15_100uMNase_Input_R1.fastq
epl1.485HA6_rep1_pt15_100uMNase_Input_R2.fastq
epl1.485HA6_rep1_tp0_100uMNase_H4K8ac_R1.fastq
epl1.485HA6_rep1_tp0_100uMNase_H4K8ac_R2.fastq
epl1.485HA6_rep2_pt15_100uMNase_H4K8ac_R1.fastq
epl1.485HA6_rep2_pt15_100uMNase_H4K8ac_R2.fastq
epl1.485HA6_rep2_pt15_100uMNase_Input_R1.fastq
epl1.485HA6_rep2_pt15_100uMNase_Input_R2.fastq
epl1.485HA6_rep2_tp0_100uMNase_H4K8ac_R1.fastq
epl1.485HA6_rep2_tp0_100uMNase_H4K8ac_R2.fastq
epl1.485HA6_rep2_tp0_100uMNase_Input_R1.fastq
epl1.485HA6_rep2_tp0_100uMNase_Input_R2.fastq
EPL1HA6_rep1_tp_pt15_100uMNase_H4K8ac_R1.fastq
EPL1HA6_rep1_tp_pt15_100uMNase_H4K8ac_R2.fastq
EPL1HA6_rep1_tp_pt15_100uMNase_Input_R1.fastq
EPL1HA6_rep1_tp_pt15_100uMNase_Input_R2.fastq
EPL1HA6_rep1_tp_pt15_Sonicated_HA_IP_R1.fastq
EPL1HA6_rep1_tp_pt15_Sonicated_HA_IP_R2.fastq
EPL1HA6_rep1_tp_pt15_Sonicated_Input_R1.fastq
EPL1HA6_rep1_tp_pt15_Sonicated_Input_R2.fastq
EPL1HA6_rep1_tp0_100uMNase_H4K8ac_R1.fastq
EPL1HA6_rep1_tp0_100uMNase_H4K8ac_R2.fastq
EPL1HA6_rep1_tp0_100uMNase_Input_R1.fastq
EPL1HA6_rep1_tp0_100uMNase_Input_R2.fastq
EPL1HA6_rep1_tp0_Sonicated_HA_IP_R1.fastq
EPL1HA6_rep1_tp0_Sonicated_HA_IP_R2.fastq
EPL1HA6_rep1_tp0_Sonicated_Input_R1.fastq
EPL1HA6_rep1_tp0_Sonicated_Input_R2.fastq
EPL1HA6_rep2_tp_pt15_100uMNase_H4K8ac_R1.fastq
EPL1HA6_rep2_tp_pt15_100uMNase_H4K8ac_R2.fastq
EPL1HA6_rep2_tp_pt15_100uMNase_Input_R1.fastq
EPL1HA6_rep2_tp_pt15_100uMNase_Input_R2.fastq
EPL1HA6_rep2_tp_pt15_Sonicated_HA_IP_R1.fastq
EPL1HA6_rep2_tp_pt15_Sonicated_HA_IP_R2.fastq
EPL1HA6_rep2_tp_pt15_Sonicated_Input_R1.fastq
EPL1HA6_rep2_tp_pt15_Sonicated_Input_R2.fastq
EPL1HA6_rep2_tp0_100uMNase_H4K8ac_R1.fastq
EPL1HA6_rep2_tp0_100uMNase_H4K8ac_R2.fastq
EPL1HA6_rep2_tp0_100uMNase_Input_R1.fastq
EPL1HA6_rep2_tp0_100uMNase_Input_R2.fastq

EPL1HA6_rep2_tp0_Sonicated_HA_IP_R1.fastq
EPL1HA6_rep2_tp0_Sonicated_HA_IP_R2.fastq
EPL1HA6_rep2_tp0_Sonicated_Input_R1.fastq
EPL1HA6_rep2_tp0_Sonicated_Input_R2.fastq
pt15_rep1_H3K23ac_R1.fastq
pt15_rep1_H3K23ac_R2.fastq
pt15_rep1_H4K12ac_R1.fastq
pt15_rep1_H4K12ac_R2.fastq
pt15_rep1_input_R1.fastq
pt15_rep1_input_R2.fastq
pt15_rep2_H3K23ac_R1.fastq
pt15_rep2_H3K23ac_R2.fastq
pt15_rep2_H4K12ac_R1.fastq
pt15_rep2_H4K12ac_R2.fastq
pt15_rep2_Input_R1.fastq
pt15_rep2_Input_R2.fastq
tp0_rep1_H4K12ac_R1.fastq
tp0_rep1_H4K12ac_R2.fastq
tp0_rep2_H4K12ac_R1.fastq
tp0_rep2_H4K12ac_R2.fastq
tsa15_rep1_H4K12ac_R1.fastq
tsa15_rep1_H4K12ac_R2.fastq
tsa15_rep2_H4K12ac_R1.fastq
tsa15_rep2_H4K12ac_R2.fastq
Untagged_100uMNase_HA_IP_R1.fastq
Untagged_100uMNase_HA_IP_R2.fastq
Untagged_100uMNase_Input_R1.fastq
Untagged_100uMNase_Input_R2.fastq

Genome browser session
(e.g. UCSC)

Please refer to wig files at https://www.ncbi.nlm.nih.gov/geo/query/acc.cgi?acc=GSE110287

Methodology

Replicates

Duplicate biological replicates were performed for each condition, except for the MNase Epl1 and Epl1(1-485) ChIP-seq experiments. For these, only one replicate was performed, but good agreement with the sonicated ChIP-seq data was used to validate these data

Sequencing depth

Sample, Mapped Reads, Uniquely Mapped Reads
EPL1-6HA_MNase_HA_IP_tp0, 13662338, 12318028
epl1.485-6HA_MNase_HA_IP_tp0, 18180146, 16784518
EPL1-6HA_Sonic_HA_IP_pt15_rep1, 7766190, 7529484
EPL1-6HA_Sonic_HA_IP_pt15_rep2, 4156226, 4084592
EPL1-6HA_Sonic_HA_IP_tp0_rep1, 4652560, 4567586
EPL1-6HA_Sonic_HA_IP_tp0_rep2, 9314654, 9035532
epl1.485-6HA_Sonic_HA_IP_pt15_rep1, 3710104, 3658112
epl1.485-6HA_Sonic_HA_IP_pt15_rep2, 3249480, 3198912
epl1.485-6HA_Sonic_HA_IP_tp0_rep1, 4777056, 4691198
epl1.485-6HA_Sonic_HA_IP_tp0_rep2, 3165104, 3126848
Untagged_Sonic_HA_IP_tp0, 7342588, 6915818
H3K23ac_pt15_rep1, 5085478, 4928616
H3K23ac_pt15_rep2, 19940100, 19058578
H3K23ac_tp0_rep1, 18627464, 17938562
H3K23ac_tp0_rep2, 16525690, 15952832
H3K23ac_tsa15_rep1, 18677944, 18037672
H3K23ac_tsa15_rep2, 15002682, 14578530
H4K12ac_pt15_rep1, 15903400, 15436774
H4K12ac_pt15_rep2, 18761686, 18219906
H4K12ac_tp0_rep1, 16730100, 16256180
H4K12ac_tp0_rep2, 14442902, 13939340
H4K12ac_tsa15_rep1, 18485180, 17820332
H4K12ac_tsa15_rep2, 15710962, 15219514
EPL1-6HA_MNase_H4K8ac_IP_pt15_rep1, 17754046, 16922200
EPL1-6HA_MNase_H4K8ac_IP_pt15_rep2, 19216428, 18133552
EPL1-6HA_MNase_H4K8ac_IP_tp0_rep1, 9262270, 8953380
EPL1-6HA_MNase_H4K8ac_IP_tp0_rep2, 19195174, 17846466
EPL1-6HA_MNase_Input_pt15_rep1, 6508738, 6327076
EPL1-6HA_MNase_Input_pt15_rep2, 10003528, 9647302
EPL1-6HA_MNase_Input_tp0_rep1, 8025918, 7709658

EPL1-6HA_MNase_Input_tp0_rep2, 11124518, 10679732
epl1.485-6HA_MNase_Input_tp0_rep1, 14416746, 13611786
MNase_Input_pt15_rep1, 16712192, 16211722
MNase_Input_pt15_rep2, 13464310, 13050912
MNase_Input_tp0_rep1, 15950112, 15478082
MNase_Input_tp0_rep2, 15947712, 15368312
MNase_Input_tsa15_rep1, 16864558, 16303470
MNase_Input_tsa15_rep2, 12664092, 12313610
epl1.485_rep1_tp0_100uMNase_Input, 16328652, 14180352
epl1.485_rep2_tp0_100uMNase_Input, 12019562, 10844740
epl1.485_6HA_rep1_tp0_H4K8ac, 17873716, 15970700
epl1.485_6HA_rep2_tp0_H4K8ac, 15830726, 14192766
epl1.485_6HA_rep1_tp_pt15_Input, 9149494, 8612328
epl1.485_6HA_rep2_tp_pt15_Input, 9361728, 8792122
epl1.485_6HA_rep1_tp_pt15_H4K8ac, 11424234, 10737326
epl1.485_6HA_rep2_tp_pt15_H4K8ac, 8608200, 8199060
101_rep1_tp0_100uMNase_Input, 10774056, 8489350
101_rep1_tp0_100uMNase_HA_IP, 8962918, 8384804
EPL1HA6_rep1_tp0_Sonicated_Input, 1051626, 1041370
EPL1HA6_rep2_tp0_Sonicated_Input, 1456172, 1440200
EPL1HA6_rep1_tp_pt15_Sonicated_Input, 777036, 769204
EPL1HA6_rep2_tp_pt15_Sonicated_Input, 753772, 747570
epl1.485_6HA_rep1_tp0_Sonicated_Input, 851202, 842922
epl1.485_6HA_rep2_tp0_Sonicated_Input, 910876, 902262
epl1.485_6HA_rep1_pt15_Sonicated_Input, 937222, 928178
epl1.485_6HA_rep2_pt15_Sonicated_Input, 1191712, 1180420

Antibodies

Anti-H3K23ac (Active Motif, 39131, lot # 1008001), anti-H4K8ac (Abcam, ab45166, clone # EP1002Y), anti-H4K12ac (Active Motif, 39165, lot # 1008001), anti-HA (Roche, 12CA5, lot # 11849700),

Peak calling parameters

No peak calling algorithms were used.

Data quality

High correspondence between replicates was confirmed by genome-wide correlation analysis.

Software

Data was collected using standard Illumina software for the NextSeq 500 and HiSeq 2500 platforms.
